# Mind the costs, too: towards better cost-effectiveness analyses of PBF programmes

**DOI:** 10.1136/bmjgh-2018-000994

**Published:** 2018-10-15

**Authors:** Y-Ling Chi, Mohamed Gad, Sebastian Bauhoff, Kalipso Chalkidou, Itamar Megiddo, Francis Ruiz, Peter Smith

**Affiliations:** 1 School of Public Health, Imperial College London, London, UK; 2 Center for Global Development, Washington, District of Columbia, USA; 3 Harvard T.H. Chan School of Public Health, Harvard University, Cambridge, MA, United States; 4 Management Science, University of Strathclyde, Glasgow, UK; 5 Imperial College Business School, London, UK

**Keywords:** performance-based financing, cost-effectiveness analysis, aid transition

**Summary box**

- The evidence surrounding the cost-effectiveness of performance-based financing (PBF) is weak, and it is not clear how PBF compares with alternative interventions in terms of its value for money.
- It is important to fill this evidence gap as countries transition from aid and face increasing budget constraints and competing priorities for the use of their domestic resources.
- In conducting cost-effectiveness analyses of PBF, researchers should be mindful of the identification, measurement and valuation of costs and effects, provide justification for the scope of their studies, and specify appropriate comparators and decision rules.
- We also recommend the use of a reference case to lay out the principles, preferred methodological choices and reporting standards, as well as a checklist.

## Introduction

In the last two decades, performance-based financing (PBF) has gained worldwide prominence. As of September 2016, the Health Results Innovation Trust Fund (HRITF) at the World Bank supported 29 low-income and middle-income countries in the introduction, implementation and evaluation of 35 PBF programmes, with expenditure near $2.5 billion. Although PBF is perceived as a tool to achieve the Sustainable Development Goals, several global health experts have pointed to its mixed evidence base.^1^ In recent years, PBF has become one of the most divisive topics in the global health community, as illustrated by the lively discussions following the publication of Paul *et al*’s piece.^2^

Policy-makers need to assess whether PBF is an appropriate policy choice for their countries, given the substantial budget constraints they face and that are likely to worsen with transition from aid. In practice, whether the investments required to implement PBF are ‘worthwhile’ is a question that can be answered by a cost-effectiveness analysis (CEA). However, a recent review found no studies making clear connections between the costs and effects of PBF.^3^ Our search yielded three CEAs of PBF; only one was peer-reviewed.^4–6^ This commentary reviews those studies and discusses potential methodological pitfalls. We also seek to offer constructive ways to move forward with undertaking robust CEA.

## How CEA can be applied to PBF

CEA synthesises the evidence on the costs and effectiveness of an intervention. The outcome of a CEA is usually the incremental cost-effectiveness ratio (ICER), which is the difference in costs between two interventions divided by the difference in their health outcomes. Health effects are often measured as averted disability-adjusted life years (DALYs) or gained quality-adjusted life years (QALYs). The main objective of CEAs is to rank interventions according to their costs and effectiveness, allowing policy-makers to select interventions that produce the greatest health at the lowest cost.^7^ In other words, CEA will show how PBF compares with other interventions, and whether investments to support PBF could produce better health outcomes than alternative spending scenarios.

Although the key ingredients of CEAs—information on cost and impact—are in principle available for a number of PBF programmes, carrying out a CEA for this kind of complex intervention is challenging. In particular, assessing the effectiveness of PBF through direct measurement or modelling can be difficult. PBF is a complex intervention that is likely to influence health outcomes through multiple and intricate channels, including spillovers from increased provider autonomy, improved health information systems, monitoring of quality of care and public finance management.^8^ There is currently little guidance on how to gauge the effectiveness for complex interventions like PBF, and no clear framework for extrapolating from effects on, for example, service utilisation and quality to health outcomes. As a result, studies use different modelling approaches that are not validated and do not align with the traditional CEA literature. This, in turn, makes it difficult to compare CEAs of PBF with those of alternative policy choices or draw meaningful conclusions on the value of PBF. For example, the CEA of the Zambia PBF programme used a combination of estimates from the Lives Saved Tool and Delphi panels to extrapolate from changes in access to essential services and quality indicators to health outcomes.^4^ The effectiveness of PBF is expressed in terms of ‘Quality adjusted QALYs’, a novel measure that cannot be compared with existing literature.

## Towards informative CEAs of PBF

We recommend the following steps towards generating CEA studies for PBF: performing systematic and transparent identification, measurement, and valuation of appropriate costs and effects, providing justification for the scope of the CEA, and deciding on a clear comparator against which PBF will be assessed and applying a relevant decision rule criterion. Although inherent trade-offs will remain, these steps are feasible and could substantially advance the base of policy-relevant evidence on PBF.

First, the costing perspective needs to be aligned with the objectives of the CEA (eg, to inform country or donor decisions) and be consistent with the costing methods. Studies using a health systems’ perspective should include all relevant costs to the health system. Typically, this requires information on activities that are often not the evaluators’ focus, such as measuring time usage patterns of providers. For instance, the Zambian PBF CEA did not account for this cost category,^4^ but it appears to be important elsewhere (eg, in the Tanzanian PBF programme, 17% of provider time was associated with entering and verifying performance data^5^). Failure to include these costs will impact the cost estimates of PBF and make it hard to compare results across studies as standard CEA practice would include such costs. Having clearly laid out methods for costing is essential to ensure that all costs are accounted for and that studies use comparable methods. The Reference Case for Estimating the Costs of Global Health Services and Interventions provides an excellent resource on this subject.^9^

A second step is to define what is meant by *effectiveness* of PBF programmes, and how to measure and/or model it. Typically, decision analytic modelling is used in CEAs to extrapolate from intermediary or short-term outputs to final and longer term outcomes (eg, health outcomes). PBF is unique as it has multiple objectives and impacts health through different direct or indirect channels. However, measuring changes in health outcomes or quality of life (eg, through household surveys) is commonly used to perform such modelling. In a CEA study of the Plan Nacer (a PBF programme) in Argentina, changes in birth outcomes and neonatal mortality were used in tandem to estimate DALYs averted.^6^ However, there is no existing clear framework for measuring or modelling the effects of complex interventions like PBF.

Special attention should also be given to the scope of the CEA. In some countries, PBF programmes are large and affect several disease areas at once, possibly over varying lengths of time. Evaluating the entirety of a PBF programme might not be possible. Moreover, PBF seems to be more effective at improving some aspects of care than others.^1^ As a result, focusing on a segment of the programme (eg, child health) and a specific exposure period might be preferable. Should the researchers decide to only focus on specific aspects, a robust justification for selecting the areas of care is needed. The decision rule should be decided from the onset,^10^ and researchers should not select areas of care where measured effectiveness is the largest and discard evidence from areas that show no impact. The decision rule in Zambia was not clear.^4^ Moreover, rigorous methods for attributing costs to the relevant segment should be applied and clearly reported. This can be challenging, especially when it comes to fixed costs. Time and motion surveys, facility surveys, and provider surveys can help with identifying costs accruing to certain services.

When possible, expected costs and effects should be considered for all relevant alternatives to PBF. For instance, some HRITF studies sought to compare PBF with unconditional cash disbursements. Another option could be to compare PBF with an approach of performance monitoring without incentive payments. When doing so, a full incremental analysis should be conducted. In other words, the estimated costs and health generated should be reported for each intervention arm, along with the corresponding ICERs. That way, the relevant cost-effective option can be identified. None of the reviewed studies presented this information clearly, which limited our ability, as readers, to clearly interpret the results.

Finally, decision rules (ie, how we decide whether an intervention is cost-effective or not) should be laid out clearly from the onset. The PBF programmes in Argentina and Zambia were judged to be cost-effective because the ICER was lower than a predefined threshold based on gross domestic product (GDP).^4 6^ While the use of a threshold is not uncommon, several experts (including at the WHO, where the practice originated) have emphasised that GDP-based thresholds are set too high and will lead to the inclusion of unaffordable interventions. Decision thresholds could be set lower^11^ (although this presents its own caveats^7^) or could be derived from available budgets.

## Conclusion

It is important to evaluate how PBF compares with other policies in terms of costs in addition to effectiveness, as country budgets are expected to take over the funding of PBF programmes in the long term.^12^

No perfect design for CEA of PBF exists, and researchers will be faced with important trade-offs. Some of those trade-offs are discussed in Shepard *et al*,^10^ and this commentary raises additional points. Planning for a CEA should be incorporated from the outset, to help define the decision problem, identify data collection needs and avoid retrospectively making up for data gaps. Methodological standards used in the general CEA literature should be followed and methods reported explicitly, allowing readers to judge the applicability of the PBF programme to their own setting and enabling comparisons and replication. Preregistration of the analysis plans, as done in clinical trials, could be used to get external comments and further strengthen the legitimacy and credibility of studies. We also strongly encourage the use of a reference case that clearly lays out principles, preferred methodological choices and reporting standards, with specific methodological discussions and specifications to accommodate the peculiarities of PBF. A starting point could be the Reference Case for Economic Evaluation developed by the International Decision Support Initiative^13^; the development of a checklist can also be envisaged.
